# Does the Arthropod Microbiota Impact the Establishment of Vector-Borne Diseases in Mammalian Hosts?

**DOI:** 10.1371/journal.ppat.1004646

**Published:** 2015-04-09

**Authors:** Constance A. M. Finney, Shaden Kamhawi, James D. Wasmuth

**Affiliations:** 1 Department of Biological Sciences, Faculty of Science, University of Calgary, Calgary, Alberta, Canada; 2 Laboratory of Parasitic Diseases, National Institute of Allergy and Infectious Diseases, National Institutes of Health, Bethesda, Maryland, United States of America; 3 Department of Ecosystem and Public Health, Faculty of Veterinary Medicine, University of Calgary, Calgary, Alberta, Canada; Stony Brook University, UNITED STATES

## Abstract

The impact of the microbiota on the immune status of its host is a source of intense research and publicity. In comparison, the effect of arthropod microbiota on vector-borne infectious diseases has received little attention. A better understanding of the vector microbiota in relation to mammalian host immune responses is vital, as it can lead to strategies that affect transmission and improve vaccine design in a field of research where few vaccines exist and effective treatment is rare. Recent demonstrations of how microbiota decrease pathogen development in arthropods, and thus alter vector permissiveness to vector-borne diseases (VBDs), have led to renewed interest. However, hypotheses on the interactions between the arthropod-derived microbiota and the mammalian hosts have yet to be addressed. Advances in DNA sequencing technology, increased yield and falling costs, mean that these studies are now feasible for many microbiologists and entomologists. Here, we distill current knowledge and put forward key questions and experimental designs to shed light on this burgeoning research topic.

## Introduction

Recent advances in DNA sequencing technologies are leading to dramatic discoveries across a broad range of biological and medical themes. For example, it is now possible to survey the microbial communities present in a wide array of ecological and biological niches. This work has led to the terms “metagenomics” and “microbiome” (Box 1) appearing frequently in both the scientific and popular press. Understandably, the focus has been on the human microbiome, with sequencing of various tissues revealing a significant diversity in composition within and between individuals. Differences in the human microbiota (Box 1) influence the immune system and are associated with various diseases, including chronic conditions such as inflammatory bowel disease, as well as infectious diseases [1]. However, comparatively little is currently known about the microbiota of invertebrates. These microbe-invertebrate interactions may be of particular relevance in aiding our understanding of vector-borne diseases (VBDs, Box 1).

**Box 1. Glossary**

**Metagenomics**

This term has been defined as “the application of modern genomics techniques to the study of communities of microbial organisms directly in their natural environments, bypassing the need for isolation and lab cultivation of individual species” [33].

**Microbiome**

Many scientific articles distinguish “microbiome” and “microbiota” to describe either the collective genomes of the microorganisms that reside in an environmental niche or the microorganisms themselves, respectively.

**Microbiota**

This term refers to both the microflora and microfauna in an ecosystem.

**Paratransgenesis**

This technique aims to decrease transmission of VBDs by eliminating pathogens from arthropod vectors through transgenesis (introduction of exogenous genes) of a vector symbiont.

**Symbiotic species**

Symbionts are species that have a long-term interaction. Specifically for this review, we refer to symbionts as commensal, mutualistic, and parasitic relationships that exist between two or more groups of organisms. They may be obligate or not.

**Vector-borne disease (VBD)**

For the purposes of this review, this term refers to infections due to pathogens transmitted by arthropod vectors.

Arthropods act as vectors for a large array of pathogens (defined here as causative agents of VBDs), transmitting them between animal or plant hosts. The direct impact on human health is considerable; tick-borne Lyme disease, as well as malaria, chikungunya, and dengue, all transmitted by mosquitoes, represent newsworthy VBDs.

Many arthropods harbor large communities of diverse microorganisms [2] that, as with human hosts, almost certainly exceed the number of cells that make up the host itself [3]. These microorganisms live within the digestive tract and/or salivary glands of arthropods where they can interact with vector-borne pathogens and/or influence their lifecycle. The microbiota and/or microbiomes within certain arthropod vectors have been extensively catalogued and reveal a large diversity (reviewed in [4]).

In mammals, pathogen loads are greatly affected by the microbiome. Similarly, vector microbiomes have been shown to naturally diminish pathogen transmission from the vector to the host by decreasing pathogen loads in the vector [5–11]. However, this is dependent on the type of bacteria (e.g., Gram + versus Gram-) [12]. These findings are a first step towards creating tools that can alter the arthropod microbiota towards reducing vector-borne pathogen transmission. Indeed, paratransgenic studies (Box 1) are focusing on transforming specific arthropod microbiota to express gene products that interfere with pathogen transmission [13]. Although this work will help curb VBD transmission, the effect of arthropod microbiota on the establishment of VBDs—once they have been transmitted to their mammalian hosts—urgently requires further investigation to help researchers understand how these microorganisms affect the mammalian host’s immune response to the vector-borne pathogen.

Despite the research to date, and the increasing amount of data generated through new sequencing techniques, we are still not at a stage where we have comprehensively studied arthropod vector microbiomes. Moreover, reports of the vector microbiomes of the same arthropods have varied, depending on factors such as life-stage [14,15], sex [16,17] and geographical distribution/sampling time [6,18–21]. Whether the differences observed so far are due to differences in research methods (Box 2) remains under investigation. An additional shortcoming is that studies have typically focused on bacteria and ignored viruses, protozoa, and fungal species, and few studies have distinguished resident from transient microbial populations [14], though symbionts (Box 1) have been observed in the salivary glands of certain vectors such as *Glossina* flies, ticks, and mosquitoes [22–24]. Additionally, most research groups sequencing vector microbiomes have concentrated on characterizing whole arthropod or gut microbiomes [14,25]. In human medicine, there is a desire to move beyond cataloguing bacteria species to consideration of their functional interactions with the host [26]; the latter is likely to result in a more robust assessment of microbe-host interactions. Highlighting potentially overlapping functions may also help pinpoint the relevant variability in the microbiome. A similar approach is essential if we are to understand the impact of the microbiota on the transmission and establishment of VBDs.

**Box 2. Methods used to study microbiomes**

Traditionally, microbial studies of arthropod vectors have had to rely on culture-based techniques and have been more interested in identifying pathogens than commensals/mutualistic species (reviewed in [4]). However, only a proportion of bacterial species grow under culture conditions, which has severely limited our knowledge of the microbial communities present in a given ecological niche. The advances in DNA sequencing technologies, termed next-generation sequencing (NGS), are now allowing the study of mixed communities. NGS is now being used to understand the microbiota of arthropod vectors.

Microbiome amplicon sequencing targets the 16S ribosomal RNA gene, in a high throughput manner, with significantly reduced bias: redundant PCR primers are designed against constant regions of the 16S rRNA gene, which flank the more variable regions. Millions of sequence reads are generated, which can then be clustered using any of a growing number of algorithms and software. The result is a clearer idea of bacterial species richness and relative abundance in arthropod vectors. However, caution must be taken in interpreting data generated by NGS. Few studies have combined culture and PCR-based approaches to characterize arthropod microbiota [34–36], even though both methods yield different, yet complementary results.

So far, microbiome projects have concentrated on bacterial elements. Indeed, these are relatively easy to identify, originally grown in culture and more recently through 16S rRNA sequencing. However, in vivo, the microbiota of arthropods is composed of bacteria, viruses, fungi, and other eukaryotic parasites. These other microbes should not be dismissed when cataloguing the microbiota. Fungal species (symbiotic yeast species and the potentially pathogenic *Aspergillus* respectively) have already been identified in the guts of *Aedes* and *Culex* mosquitoes [37,38]. These could impact VBD transmission just as much as symbiotic bacteria, such as the Enterobacteriaceae family for malaria [6]. It will be crucial to discriminate between microbes that are resident, i.e., relatively static in a niche, and those that are transient, which may play the most important role in transmission and establishment of a VBD. Here, alternative technologies can provide additional and supporting information. For example, fluorescent in situ hybridization (FISH), reveals the location and abundance of a targeted microorganism [39]. Abundance data is considerably harder to determine with 16S sequencing because of dramatic gene copy number variation even between relatively closely related species; within the genus *Bacillus* the 16S copy number of different species varies between seven and 14 [40].

Noting the type of symbiont identified is also paramount. Commensal, mutualistic, and parasitic species may have very different impacts on VBD transmission. Indeed the impact of commensals of humans on the human host has been shown to depend on the density of the species: beneficial at some levels, harmful at others [41]. Also, the transmission of the bacterial species between vectors (horizontal or vertical) will affect how useful they are as a tool in curbing VBD transmission. Indeed, obligate species like *Wolbachia*, which are parasitic in many insects, and which are transmitted vertically have already been introduced into vector populations and shown to reduce VBD transmission and remain stable in the vector population [42]. Even genotypes of the same strain can have different impacts on VBD transmission. Differences in genotypes of *Sodalis*, a symbiont of the tsetse fly, result in differences in transmission of trypanosomes [43].

At this point, our knowledge of vector microbiomes is still limited and consists mostly of an emerging appreciation of their impact on pathogens carried by the vectors. Research has so far only focused on the role of the vector microbiota within their arthropod host, where they can strongly influence the transmission of vector-borne pathogens [5–11].

**Fig 1:**
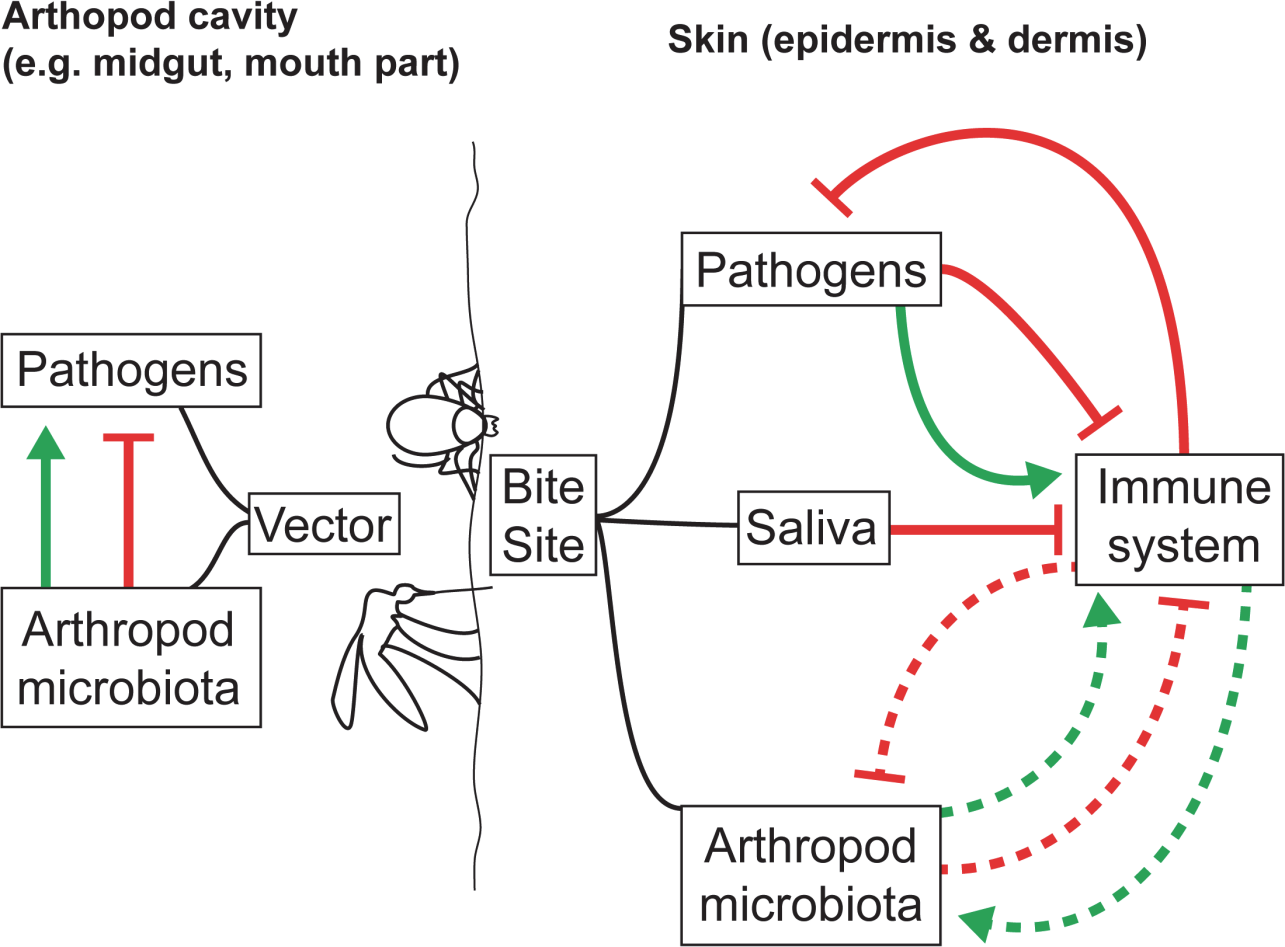
How arthropod microbiota could enhance/interfere with the transmission/establishment of VBDs. In the arthropod cavity, the arthropod microbiota can alter pathogen development, resulting in decreased or increased loads in the vectors and reduced or increased transmission. However, the impact of the pathogens on the microbiota has yet to be assessed. Once transmission has occurred, the host immune system generates a response to destroy the pathogens in the skin. Components from the pathogens themselves and the arthropod saliva are known to actively inhibit this process. The role of the arthropod microbiota, likely transmitted along with the pathogens, on the host immune system is currently unknown (dotted lines).

What is entirely missing is an understanding of how vector microbiomes may affect the immune response of the mammalian host to the pathogen following the bite of an infected arthropod. The impact of vector microbiota on skin-mediated host immunity and on the establishment of VBDs within the mammalian host have yet to be investigated in any depth, despite direct relevance to disease control (Fig 1). Infection transmission routes make the transmission of vector-borne microbiota along with vector-borne pathogens likely. Injection (e.g., ticks, fleas, mosquitoes, sand flies) could lead to the transmission of salivary gland and gut microbiomes through feeding and regurgitation cycles. A number of vector salivary components delivered to the host during arthropod feeding strongly impact VBD dynamics and outcome [27]. If transmitted through saliva or co-deposited with the pathogen and saliva, could the vector microbiome impact downstream host immune responses to the arthropod bite and any vector-borne pathogens? Since the mammalian and vector microbiomes can stimulate the immune system of their hosts [8,7,28], this seems likely. The composition of the biofilm growth of *Yersinia pestis* transmitted to the mammalian host is thought to contain flea gut and bacterially derived components [29]. Defecation (e.g., for reduviids, which transmit Chagas disease–causing trypanosome parasites) could also lead to the transmission of gut microbiome components to the vertebrate host.

Establishing the transfer of vector microbiota to the host represents an important step towards understanding the initial host immune response to arthropod bites and vector-borne pathogens. As a first step, available genomic and transcriptomic data from vector saliva or midguts (e.g., ticks [30] and sand flies [31]) can be used to identify the presence of vector microbiota in arthropod saliva or midgut tissue. Once key species are identified in the vector, their presence can be confirmed at the bite site. This is a crude, indirect method, in which the host skin microbiome may act as a confounder, but it serves as a preliminary indication of the presence of vector microbiota in arthropod bites. For a more in-depth investigation, fluorescently tagged species can be introduced into the vector salivary glands and/or midguts. After a feed, fluorescence can be tracked in the host. If the microbiota are found to be transferred from vector to host, using germ-free arthropods (through temporary [9,8,7,10] or permanent removal of microbes [32]) may provide insights into whether the immune response to VBDs is modulated by the vector microbiota.

We are only just beginning to uncover the full impact of the vector microbiota on VBD disease dynamics in arthropod vectors. Their impact on the disease transmission process and mammalian immune responses to vector-borne pathogens (Fig 1) are topics that have been under-studied and largely ignored until now. However, with DNA sequencing approaches now generating huge volumes of data with which to study vector microbiomes, a new wealth of data is becoming available. It will need to be carefully collected, compiled, and analyzed in order to generate meaningful interpretations that can be applied to understanding and perhaps controlling VBDs.
